# Swallowing‑induced spontaneous pneumomediastinum after ingestion of a large, unchewed food bolus in a young adult: a case report

**DOI:** 10.1186/s12245-026-01273-3

**Published:** 2026-07-03

**Authors:** Motoyasu Nakamura, Keisuke Suzuki, Eriko Yoshida, Yoshimi Miyamoto, Tomohiro Nomoto, Kenji Dohi, George Wada

**Affiliations:** 1 Department of General Medicine, Kawasaki Kyodo Hospital, Kawasaki Medical Cooperative Association, Kawasaki, Japan; 2 Department of Emergency, Critical care and Disaster Medicine, School of Medicine, Showa Medical University, Tokyo, Japan; 3 Department of Gastroenterology, Kawasaki Kyodo Hospital, Kawasaki Medical Cooperative Association, Kawasaki, Japan

**Keywords:** Spontaneous pneumomediastinum, Swallowing, Esophageal injury, Tracheal injury

## Abstract

**Background:**

Spontaneous pneumomediastinum (SPM) is a rare condition with a generally favorable prognosis, although severe complications, including respiratory arrest or tension emphysema, are reported; swallowing may be an under-recognized precipitating factor.

**Case presentation:**

A man in his 20s swallowed one‑third of a 10‑cm steamed meat bun without chewing. He developed throat tightness and chest pain without cough and presented to our emergency department. Chest computed tomography (CT) findings revealed extensive mediastinal emphysema; swallowing‑triggered spontaneous pneumomediastinum was diagnosed, and intravenous piperacillin/tazobactam was initiated. Esophagography on day 2 and endoscopy on day 3 showed no leakage or perforation. A CT scan on day 8 revealed near resolution, and he was discharged on day 9 without recurrence. We speculated that the large, unchewed bolus caused transient airway obstruction, leading to a sudden increase in intratracheal pressure and resulting in tracheal or alveolar microinjury.

**Conclusions:**

This case highlights swallowing of a large, unchewed food bolus as a potential trigger of pneumomediastinum. Considering swallowing‑triggered spontaneous pneumomediastinum in patients presenting with chest pain after eating may facilitate early diagnosis and help avoid unnecessary invasive investigations.

## Background

Spontaneous pneumomediastinum (SPM) is free mediastinal air without an evident cause. SPM can be subdivided into truly idiopathic cases without any identifiable trigger and those associated with precipitating events such as cough, vomiting, or strenuous activity [1]. Its incidence is estimated at < 1:44,000, occurring predominantly in young men, and it is usually benign [2, 3]. However, severe presentations such as respiratory arrest and tension pneumomediastinum have been described [4]. Asthma has been identified as a common predisposing condition in patients with SPM, being reported as an underlying comorbidity in approximately one fifth of cases in recent series [5]. The “Macklin effect,” where alveolar rupture causes air to spread into the mediastinum via the interstitium, is widely recognized as the underlying mechanism [6]. However, cases triggered by everyday swallowing are very rare. We report a rare case of SPM occurring immediately after a large, unchewed food bolus was swallowed. This report describes a rare case of spontaneous pneumomediastinum occurring immediately after swallowing a large, unchewed food bolus, without preceding cough or vomiting, highlighting swallowing as a potential but under-recognized trigger.

## Case presentation

A man in his 20s with asthma and atopic dermatitis presented with chest pain. He had no medical history of tobacco, alcohol, or drug use, and no known allergies or medication use. Earlier on the day of admission, he had swallowed one‑third of a 10‑cm steamed meat bun without chewing, after which he developed chest pain and throat tightness unrelieved by water.

On admission, his vital signs were as follows: Glasgow Coma Scale score, 15 (E4V5M6); blood pressure, 118/78 mmHg; heart rate, 98 beats/min; respiratory rate, 12 breaths/min; temperature, 37.1 °C; oxygen saturation, 98% room air. Examination revealed subcutaneous crepitus in the neck and a positive Hamman’s sign. His heart sounds were normal, lungs were clear, and the abdomen was soft and non-tender, without guarding or rebound. There was no jugular venous distension or peripheral edema. Electrocardiography showed sinus rhythm without ischemic changes. Laboratory findings demonstrated mild leukocytosis and elevated C-reactive protein levels.

**Fig. 1:**
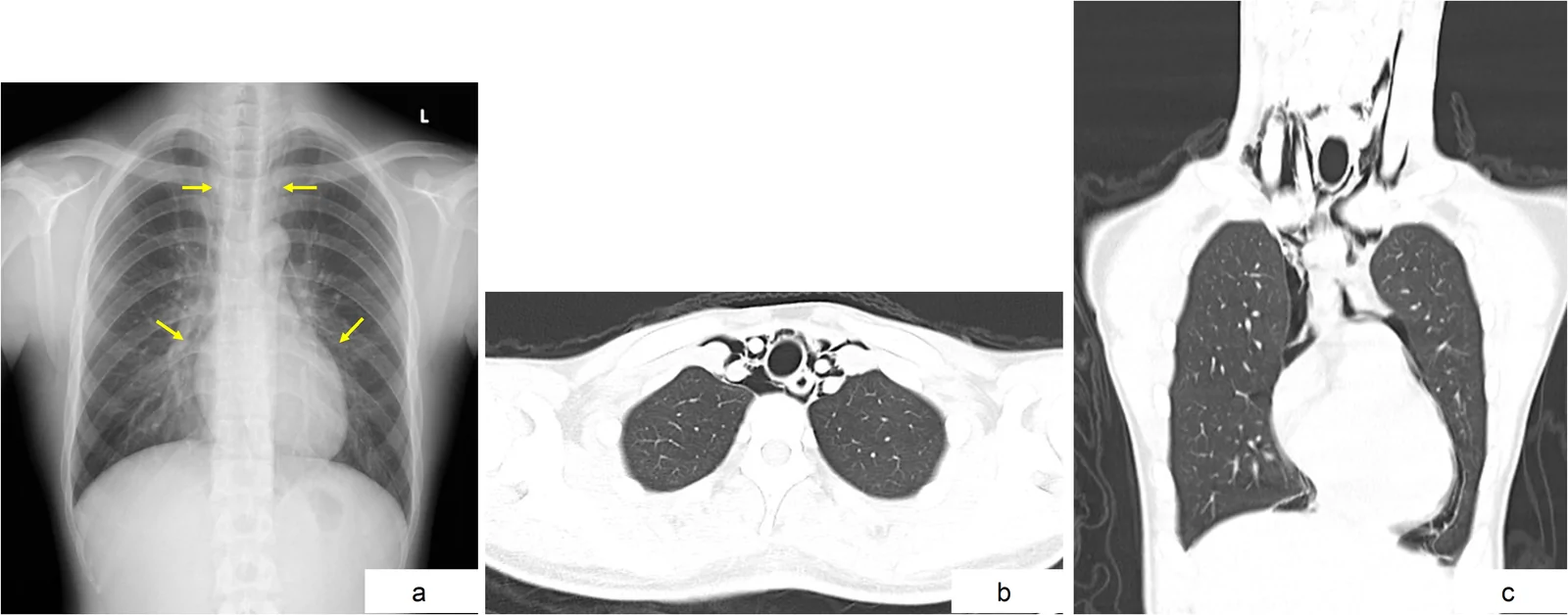
Imaging tests performed at the time of visit. **a**: Chest radiograph reveals linear luminal areas around the heart and trachea, circumscribing the mediastinum. **b**, **c**: Chest CT reveals gas from the neck to the mediastinum, but no obvious damage to the main bronchi or trachea was noted

Chest radiography indicated linear translucency along the cardiac border (Fig. 1a). Computed tomography (CT) demonstrated extensive emphysema extending from the neck to the mediastinum without perforation or pneumothorax (Figs. 1b, c). He was admitted for close observation and further evaluation to exclude esophageal or airway injury, and fasting with intravenous piperacillin/tazobactam 4.5 g every 8 h was initiated.

**Fig. 2:**
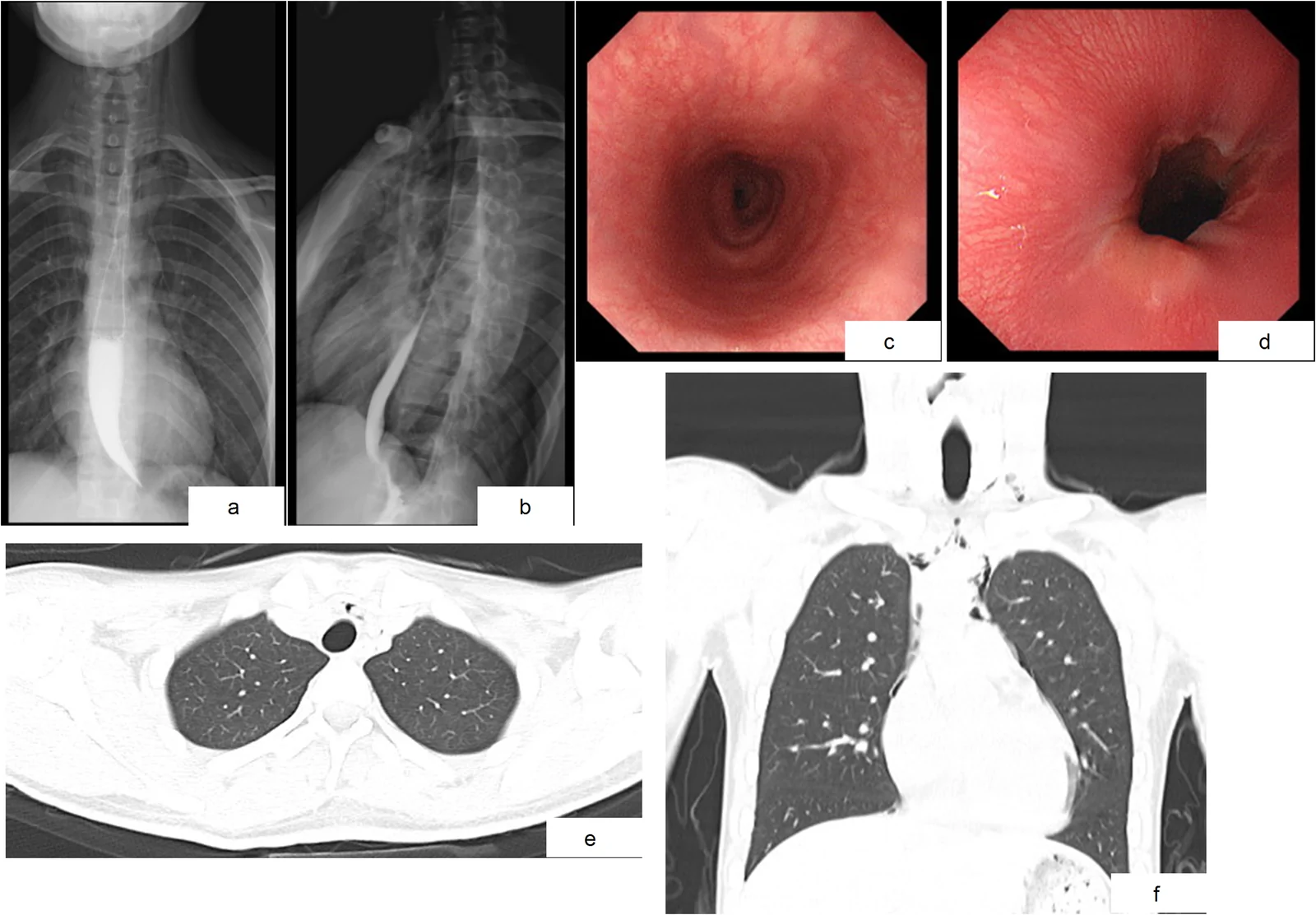
Imaging tests performed after hospitalization. **a**: Esophagogram from the second day of hospitalization, frontal view; no obvious esophageal leakage was noted. **b**: Esophagogram on the second day of hospitalization, lateral view; no obvious esophageal leakage was noted. **c**, **d**: Upper gastrointestinal endoscopy from hospital day 3; no obvious esophageal damage was noted. **e**, **f**: Neck and chest CT from hospital day 8 reveal a decrease in mediastinal emphysema

Esophagography on hospitalization day 2 and an upper endoscopy on day 3 showed no contrast leakage, mucosal disruption, or perforation, and were interpreted as normal (Figs. 2a–d). During fasting, the patient received maintenance intravenous fluids containing glucose and electrolytes, without enteral or parenteral nutrition, as short-term fasting was considered sufficient. By day 4, his pain had improved and oral intake was resumed. CT findings on hospital day 8 confirmed resolution of the emphysema (Figs. 2e, f). Antibiotics were discontinued, and he was discharged on day 9 in good condition. No recurrence occurred during a follow-up period of 12 months in the outpatient clinic.

## Discussion

In this case, a young man developed spontaneous pneumomediastinum immediately after swallowing a large, unchewed food bolus, without preceding cough, vomiting, or trauma. The temporal relationship and absence of other triggers suggest that abrupt pressure changes during swallowing may have contributed to the development of pneumomediastinum. Swallowing a large, insufficiently chewed bolus may have transiently obstructed the upper airway, causing a sudden increase in intratracheal pressure. Although CT scans did not demonstrate a typical Macklin-like radiological sign as described in recent systematic reviews, transient airway overpressure during swallowing may have caused microscopic alveolar or airway injury, with air subsequently tracking into the mediastinum via a mechanism analogous to the Macklin effect. Recent work has emphasized that a Macklin-like radiological sign, characterized by linear air collections along the bronchovascular bundles, can be identified on CT in many patients with barotrauma; however, such a sign was not evident in our patient [7]. Thus, even swallowing can create localized Valsalva-like pressure changes depending on bolus size and speed.

Spontaneous pneumomediastinum should be differentiated from serious conditions such as esophageal perforation, tracheal injury, and mediastinitis [8]. Radiography and CT are useful, but bronchoscopy, endoscopy, and esophagography should be performed selectively [9]. For suspected esophageal perforation, fluoroscopic esophagography is necessary. If there is history of trauma, ventilation, or intubation, a bronchoscopy should be considered [8]. 

Esophageal perforation, including Boerhaave syndrome, represents the most critical differential diagnosis in patients presenting with acute chest pain and pneumomediastinum. Typical features include a history of forceful vomiting or retching, sudden severe chest or upper abdominal pain, systemic toxicity, and rapid progression to mediastinitis with pleural effusion and hemodynamic instability. In our patient, there was no preceding vomiting or abdominal pain, and he remained hemodynamically stable without signs of sepsis or pleural effusion on serial CT scans. Furthermore, contrast esophagography on day 2 and upper endoscopy on day 3 demonstrated no evidence of leakage, mucosal disruption, or perforation, making esophageal perforation, including Boerhaave syndrome, highly unlikely.

Tracheobronchial injury is another important consideration in patients with extensive pneumomediastinum, particularly following blunt chest trauma, penetrating injury, endotracheal intubation, or positive‑pressure ventilation. Such injuries often present with respiratory distress, hemoptysis, and persistent large air leaks, and are commonly associated with pneumothorax that is refractory to chest tube drainage. In the present case, there was no history of trauma, intubation, or mechanical ventilation, and the patient had no dyspnea, hemoptysis, or pneumothorax on CT. CT did not reveal focal airway disruption or irregularity, and the pneumomediastinum resolved completely with conservative management, which argues against clinically significant tracheobronchial injury; therefore, bronchoscopy was not deemed necessary in this clinically stable patient.

Asthma‑related barotrauma, including pneumomediastinum secondary to severe bronchospasm, also needed to be considered given the patient’s history of asthma.

However, at presentation he had no wheezing, dyspnea, or increased work of breathing, and his oxygen saturation was normal on room air. There was no recent history of asthma exacerbation or excessive use of inhaled bronchodilators, and physical examination and CT did not reveal findings suggestive of acute asthma or air‑trapping. Therefore, asthma‑related barotrauma was considered unlikely, and the close temporal association with swallowing a large, unchewed food bolus was regarded as the most plausible trigger of spontaneous pneumomediastinum in this case.

Nevertheless, recent reports have highlighted that in the setting of acute asthma exacerbation, spontaneous pneumomediastinum and even severe subcutaneous emphysema can often be managed conservatively with appropriate treatment of the underlying asthma and close observation, as illustrated by Aljohani et al. in a pediatric case [10]. 

In this case, as the symptoms emerged following swallowing, an esophageal injury was suspected. However, esophagography and endoscopy ruled out injury, and CT showed no airway abnormality leading to spontaneous pneumomediastinum.

Several reports have described pneumomediastinum occurring after swallowing-related events, such as ingestion of fruit seeds or meat with bone, or postprandial belching, and most patients had favorable outcomes with conservative management (Table 1) [11, 12]. None involved trauma, drug use, coughing, or vomiting. Pressure changes during swallowing or postprandial states appear to be sufficient causes, with favorable outcomes observed under conservative treatment. Swallowing-related events, particularly ingestion of large or poorly chewed food boluses, may act as pressure-mediated triggers for pneumomediastinum in selected patients.

**Table 1:** Cases of diet-related spontaneous emphysema

No.	First author	Age	Sex	Medical history	Smoking	Cause	Size	Treatment	Transcription
1	Cho TJ [11]	16	Male	None	None	Peach seed eating	Not specified	Conservative treatment	Good
2	Cho TJ [11]	19	Female	appendectomy	None	boned rib of pork eating	Not specified	Conservative treatment	Good
3	Treharne C [12]	15	Male	None	None	Burping after meals	Not specified	Conservative treatment	Good

In addition to swallowing‑related events, pneumomediastinum can be associated with other rare but potentially dangerous air distributions.

Uysal et al. reported a case of pneumomediastinum accompanied by pneumocephalus and bilateral pneumo‑orbita after high‑pressure air injury, emphasizing that extensive air dissection involving the mediastinum, intracranial space, and orbits can occur but may still have a favorable prognosis with careful monitoring and conservative management [13]. 

Additionally, previous research findings have indicated predominance among young males, suggesting possible anatomical or physiological predispositions, such as airway diameter or alveolar fragility. However, case accumulation and further study are needed. Moreover, bronchoscopy was not performed in our clinically stable patient, in line with previous reports in which bronchoscopy was often omitted or did not clearly identify the exact site of air leakage [14]. 

This report highlights that swallowing a large, unchewed food bolus can precipitate pneumomediastinum in an otherwise healthy young adult. Including spontaneous pneumomediastinum in the differential diagnosis of chest pain after eating may facilitate early diagnosis and avoid unnecessary invasive testing. Emergency physicians should remain alert, even when symptoms are minor. Future research should clarify contributing factors, including bolus size, mastication, and anatomical susceptibility.

This case represents a rare example of spontaneous pneumomediastinum temporally associated with swallowing a large, unchewed food bolus in the absence of typical precipitating factors such as cough or vomiting. We speculate that the large unchewed bolus caused transient upper airway obstruction and a sudden rise in intrathoracic pressure, leading to mediastinal emphysema through microscopic airway or alveolar injury, possibly via a mechanism analogous to the Macklin effect. When chest pain or throat tightness develops shortly after swallowing—particularly a large bolus—spontaneous pneumomediastinum should be considered, even in the absence of cough or trauma, to enable timely imaging and appropriate conservative management.

## Data Availability

No datasets were generated or analysed during the current study.

## Declarations

### Authors’ information

MN, EY, YM, and GW are physicians in the Department of General Medicine at Kawasaki Kyodo Hospital, Kawasaki, Japan. KS and KD are emergency and critical care physicians at the Department of Emergency and Disaster, Critical Care Medicine, Showa Medical University School of Medicine, Tokyo, Japan. TN is a gastroenterologist at the Department of Gastroenterology, Kawasaki Kyodo Hospital, Kawasaki, Japan.

## Abbreviations

SPM: Spontaneous pneumomediastinum
CT: Chest computed tomography
